# Treatment of Acute Pelvic Inflammatory Disease

**DOI:** 10.1155/2011/561909

**Published:** 2011-12-20

**Authors:** Richard L. Sweet

**Affiliations:** Department of Obstetrics and Gynecology, University of California, Davis, CA 95817, USA

## Abstract

Pelvic inflammatory disease (PID), one of the most common infections in nonpregnant women of reproductive age, remains an important public health problem. It is associated with major long-term sequelae, including tubal factor infertility, ectopic pregnancy, and chronic pelvic pain. In addition, treatment of acute PID and its complications incurs substantial health care costs. Prevention of these long-term sequelae is dependent upon development of treatment strategies based on knowledge of the microbiologic etiology of acute PID. It is well accepted that acute PID is a polymicrobic infection. The sexually transmitted organisms, *Neisseria gonorrhoeae* and *Chlamydia trachomatis*, are present in many cases, and microorganisms comprising the endogenous vaginal and cervical flora are frequently associated with PID. This includes anaerobic and facultative bacteria, similar to those associated with bacterial vaginosis. Genital tract mycoplasmas, most importantly *Mycoplasma genitalium*, have recently also been implicated as a cause of acute PID. As a consequence, treatment regimens for acute PID should provide broad spectrum coverage that is effective against these microorganisms.

## 1. Introduction

Pelvic inflammatory disease (PID) is a spectrum of upper genital tract infections that includes endometritis, salpingitis, tuboovarian abscess, and/or pelvic peritonitis [1]. Typically, acute PID is caused by ascending spread of microorganisms from the vagina and/or endocervix to the endometrium, fallopian tubes, and/or adjacent structures [1–3]. Acute salpingitis is the most important component of the PID spectrum because of its impact on future fertility [3].

PID is one of the most frequent and important infections that occur among nonpregnant women of reproductive age and remains a major public health problem [4–8]. Among women, it is the most significant complication of sexually transmitted diseases/infections. Unfortunately, women who acquire acute PID are at risk for long-term sequelae including tubal factor infertility, ectopic pregnancy, chronic pelvic pain, and recurrent PID [9–13]. In addition, the estimated annual health care cost for PID and its complications in the United States is over $2 billion [7].

Currently, an estimated 770,000 cases of acute PID are diagnosed annually in the United States. A recent analysis by the Centers for Disease Control and Prevention (CDC) of trends in the incidence of PID demonstrated that from 1985 to 2001 rates of both hospitalized and ambulatory cases of acute PID declined (68% and 47%, resp.) [6]. This good news is mitigated by two factors. Recently, subclinical PID has been recognized as an important entity which is common among women with lower genital tract infections, especially *Chlamydia trachomatis, Neisseria gonorrhoeae*, and bacterial vaginosis (BV) [14, 15]. Subclinical PID is as likely as clinically recognized acute PID and is responsible for a greater proportion of PID-related sequelae than clinically recognized disease [16]. Secondly, is concern that the continued increases in *C. trachomatis* infections reported by the CDC in the United States will be associated with an increase in both clinical and subclinical PID.

Over the past 25 years, important advances have occurred in understanding the etiology, pathogenesis, and treatment of acute PID. As a result, major paradigm shifts have occurred in our approach to the treatment of acute PID. In the past PID was believed to be a monoetiologic infection, primarily caused by *Neisseria gonorrhoeae*. Today, the polymicrobic etiology of PID is well established and has led to utilization of broad spectrum antimicrobial regimens for treatment of acute PID [1, 2, 17, 18].

## 2. Etiology of PID

Prevention of the significant long-term complications associated with PID requires development of effective treatment strategies. Such treatment regimens are dependent upon an understanding of the microbiologic etiology of acute PID. However, elucidation of the etiology of PID has been hindered by several factors. Firstly, most studies have utilized specimens obtained from the lower genital tract (primarily cervix) and not the upper genital tract (endometrial cavity, fallopian tubes) which is the actual site of infection. Secondly, most investigations primarily focused on the sexually transmitted pathogens *N. gonorrhoeae* and/or *C. trachomati,s *and few studies have assessed the role of non-STD pathogens, especially anaerobic bacteria. Thirdly, even fewer investigations have addressed the putative role of *Mycoplasma genitalium *in the etiology of PID.

**Figure 1:**
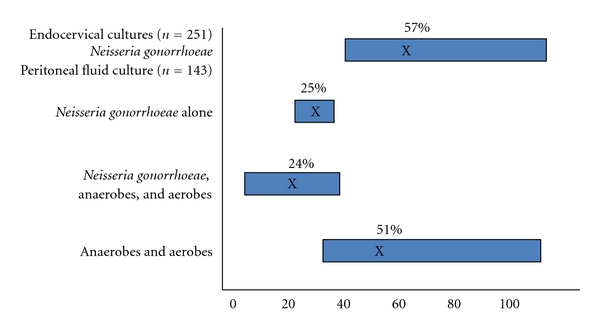
Microbiologic Etiology of Acute PID as determined by Culdocentesis, (based on references [20–25]).

PID results from the intracannicular ascending spread of microorganisms from the cervix and/or vagina into the upper genital tract. Prior to the mid-1970s, PID was believed to be a monoetiologic infection due primarily to *N. gonorrhoeae*. Based initially upon culdocentesis studies of peritoneal fluid (Figure 1) and subsequently studies utilizing laparoscopy and/or endometrial aspirations to obtain specimens from the upper genital tract (Table 1) came the recognition that the etiology of acute PID is polymicrobic with a wide variety of microorganisms involved [1, 2, 19–41]. Included among these are *N. gonorrhoeae, C. trachomatis*, genital tract mycoplasmas (particularly *M. genitalium)*, anaerobic and aerobic bacteria which comprise the endogenous vaginal flora (e.g., *Prevotella* species, black-pigmented Gram-negative anaerobic rods, *Peptostreptococci* sp., *Gardnerella vaginalis, Escherichia coli, Haemophilus influenzae*, and aerobic streptococci). 

**Figure 2:**
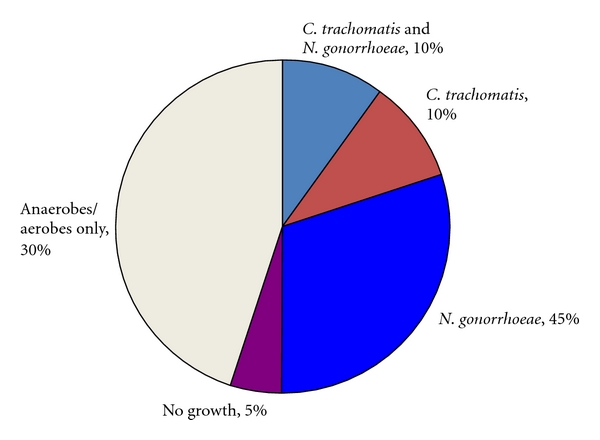
Microbiology of acute PID.

Investigations by our group conducted in the 1980s that utilized laparoscopy and/or endometrial aspirations to obtain upper genital tract specimens demonstrated that approximately two-thirds of acute PID cases were associated with *N. gonorrhoeae* and/or *C. trachomatis* (Figure 2). In nearly one-third only anaerobic and aerobic bacteria are recovered. In addition, half of the women with *N. gonorrhoeae* and/or *C. trachomatis* had concomitant anaerobic and/or aerobic bacteria recovered. More recently, in the Pelvic Inflammatory Disease Evaluation and Clinical Health (PEACH) study, the largest treatment trial of mild to moderate acute PID in the US, *N. gonorrhoeae* and *C. trachomatis* were recovered in less than one-third of patients [42].

Many of the nongonococcal, nonchlamydial microorganisms recovered from the upper genital tract in acute PID are similar to those associated with bacterial vaginosis (BV), a complex perturbation of the vaginal flora leading to loss of hydrogen peroxide producing lactobacillus and overgrowth of *G. vaginalis, Prevotella* sp. (especially *P. bivius, P. disiens*, and *P. capillosus*), *Mobiluncus* sp., black-pigmented anaerobic Gram-negative rods, alpha-hemolytic streptococci, and mycoplasmas [43]. Multiple investigations have demonstrated an association between BV and acute PID [31, 35, 43–51]. In addition, use of a broad-range 16SrDNA polymerase chain reaction to identify uncultivable bacteria has identified bacterial 16S sequences of anaerobic bacteria associated with BV in the fallopian tube of women with laparoscopically confirmed acute PID [52].

Although *M. genitalium *was identified in the early 1980s as a cause of nongonococcal urethritis in men, its role in genital tract infections in women remained unclear, due in large part to difficulty in culturing this organism. With the advent of polymerase chain reaction (PCR) technology, *M. genitalium *has been associated with cervicitis [53, 54] and has been demonstrated as an etiologic agent in nongonococcal nonchlamydial PID [36–39]. Haggerty et al. detected *M. genitalium *in 15% of women in the PEACH study [40], a rate similar to that seen in UK women (13%) [37] and west African women (16%) [36]. These rates of *M. genitalium *are similar to those seen for *C. trachomatis *and *N. gonorrhoeae *in the PEACH study of urban women in the United States. A recent analysis from the PEACH study noted that rates of short-term failure (persistent endometritis and pelvic pain), infertility, recurrent PID, and chronic pelvic pain were high among women with endometrial *M. genitalium *at baseline [40]. Subsequently, it has been demonstrated that women with *M. genitalium *infection (similar to those with chlamydial infection) present with fewer clinical signs and symptoms of acute PID than those with gonococcal infection [41]. A pathogenic role of *M. genitalium *in PID is further supported by studies demonstrating that *M. genitalium *induces salpingitis in experimental monkey studies [55] and adheres to human fallopian tube epithelial cells, in organ culture, causing damage to ciliated cells [56].

Recent attention has focused on subclinical PID. This term was initially applied to women with documented tubal factor infertility associated with evidence of chronic inflammatory residua characteristic of PID who denied a history of being diagnosed or treated for acute PID [15]. Preliminary work by our group has suggested that the microorganisms (e.g., *N. gonorrhoeae, C. trachomatis,* and bacterial vaginosis) associated with subclinical PID are the same putative agents recovered from women with clinically apparent acute PID [14].

## 3. Treatment Concepts

The therapeutic goals for treatment of acute PID include both short-term outcomes such as clinical cure and microbiologic cure and preventions of long-term sequelae such as infertility, ectopic pregnancy, recurrent infection, and chronic pelvic pain. Although the incidence rates of PID have declined, no reduction in the adverse reproductive outcomes associated with PID (infertility, ectopic pregnancy, and chronic pelvic pain) has been demonstrated [17].

While some antibiotic regimens have been successful in producing initial clinical and microbiologic cure with short-term followup, only a few studies have determined the efficacy of these treatment regimens for eliminating endometrial or fallopian tube infection. In addition, few studies have attempted to assess the incidence of long-term sequelae (e.g., tubal factor infertility, ectopic pregnancy and chronic pelvic pain) following treatment with these antibiotic regimens [1, 10, 11, 42].

In the preantibiotic era most cases of acute PID managed by conservative supportive care resolved spontaneously with studies demonstrating that approximately 85% of patients with acute PID improved clinically without the need for surgical intervention. The other 15% had prolonged or progressive symptoms requiring surgical intervention. In addition, there was approximately a 1% mortality rate. The introduction of antibiotics into clinical practice led to improvement in the prognosis for acute PID, and mortality was nearly eliminated. Studies assessing fertility rates following acute PID showed a general improvement in fertility with the mean pregnancy rate increasing from 27.9% (range 24%–43%) in the preantibiotic era to 73.1% (range 24%–81%) in the post-antibiotic era [57]. While this finding is satisfying, these results are still far from adequate.

As reviewed above, PID is a polymicrobial infection. According to the CDC, PID treatment regimens must provide broad spectrum coverage of likely pathogens [1]. Substantial evidence supports the role of *N. gonorrhoeae, C. trachomatis*, anaerobic bacteria, and facultative bacteria in the pathogenesis of acute PID [1–5, 9]. Not only are *N. gonorrhoeae* and *C. trachomatis* frequently recovered from the upper genital tract in women with PID, excellent data demonstrates the role these pathogens play in producing tubal damage and in the development of the adverse sequelae of PID (e.g., infertility, ectopic pregnancy) [57–60]. Thus, antimicrobial regimens for the treatment of acute PID must be effective against these STD organisms. While some antimicrobial regimens that do not provide adequate coverage against *N. gonorrhoeae* and/or *C. trachomatis* have been shown to have excellent clinical cure rates, microbiologic cure rates are less impressive (or lacking), and long-term outcome data are not available [17, 18, 61–64]. The CDC in its 2010 treatment recommendations [1] notes that all regimens used to treat acute PID should provide adequate coverage against *N. gonorrhoeae* and *C. trachomatis*, as they are both commonly present and have the propensity to produce tubal damage directly (*N. gonorrhoeae*) or indirectly via the host immune response (*C. trachomatis*).

The putative role of nongonococcal nonchlamydial bacteria, especially anaerobes and more recently *M. genitalium*, in the pathogenesis of acute PID and whether antimicrobial regimens for treatment of PID should provide coverage against these microorganisms is more controversial. Some propose that anaerobic coverage is only required in patients with severe PID [2], especially those with tuboovarian abscesses. Others suggest that anaerobic coverage should be provided to all women with acute PID [1]. Clearly anaerobic bacteria have been demonstrated in the upper genital tract of women with acute PID with anaerobic bacteria recovered from the upper genital tract in 13% to 78% of women with PID [28–35]. In addition, anaerobes (e.g., *Bacteroides fragilis*) have caused tubal damage in vitro studies [1].

Bacterial vaginosis (BV) has been noted to be frequently present in women presenting with acute PID [1, 43, 51]. In the PEACH study, two-thirds of the women had concomitant BV [45]. Moreover, in the PEACH study women with acute endometritis on endometrial biopsy were commonly infected with BV-associated microorganisms in their upper genital tract (*G. vaginalis* 30.5%, anaerobic Gram-negative rods 31.7%, and anaerobic Gram-positive cocci 22%) [45]. Multiple previous studies [31, 43–49] support the findings of the PEACH study conclusions that BV is associated with acute PID. In addition, the Gyn Infectious Follow-through (GIFT) study, a longitudinal study of women with BV, demonstrated that the presence of BV-related microorganisms significantly increased the risk for acquiring PID [65].

The PEACH Study authors concluded that BV-associated organisms are very commonly present in women with mild-to-moderately severe PID and suggested that treatment regimens for all women with PID include antimicrobial agents effective against anaerobes associated with BV. In a similar vein, the CDC notes that until treatment regimens that do not adequately cover these BV-associated anaerobes have been demonstrated in clinical trial to prevent the long-term sequelae of PID as efficaciously as regimens which provide effective coverage for these microbes, use of regimens with antianaerobic activity should be considered.

Limited data suggest that failure to cover anaerobes in women with acute PID may predispose them to development of long-term sequelae. In the 1970s when single agent monotherapy was the standard for treatment of PID, Chow et al. noted that tuboovarian abscesses developed in PID patients being treated solely with tetracycline [19]. Subsequently, our group reported that anaerobic bacteria persisted in the endometrial cavities of women with PID treated with ciprofloxacin despite apparent clinical cure [62]. This finding is analogous to the finding by our group that failure to include an antimicrobial agent effective against *C. trachomatis* resulted in persistent chlamydial infection in the endometrial cavity [61]. In a proof of concept study, Eckert and coworkers demonstrated that women at high risk for PID but without a clinical diagnosis of PID improved with antimicrobial regimens that provided anaerobic coverage as measured by clinical improvement and resolution of histologic endometritis [66].

Neither the 2010 CDC sexually transmitted disease treatment guidelines [1] nor the 2007 European guideline for management of pelvic inflammatory disease [2] strongly advocate for anaerobic coverage in the treatment of acute PID. However, because of the substantial evidence that anaerobes are commonly recovered from women with mild-to-moderate and severe PID, and that failure to eradicate anaerobes from the upper genital tract may lead to tubal damage, it seems prudent to do so. Firstly, as noted above, until those regimens that do not provide adequate anaerobic coverage have been shown to prevent adverse sequelae as well as those that do, it seems advisable to provide anaerobic coverage. A second strong reason for providing anaerobic coverage is the frequent (up to 70%) occurrence of BV in women with PID [50]. Thirdly, anaerobes are widely recognized as important pathogens in severe PID [67]. Severe PID, as determined by laparoscopy, not clinically, is an important determinant of future infertility [10, 68]. Thus, unless severe tubal disease has been excluded at laparoscopy, coverage for anaerobes may have important implications for the future reproductive health of these women.

On the other hand, reservation regarding the need for anaerobic coverage for acute PID has been raised. The PEACH trial [42] compared inpatient with outpatient treatment regimens in which patients were randomized to intravenous cefoxitin and doxycycline for a minimum of 48 hours (followed by oral doxycycline for a total of 14 days) or to a single dose of cefoxitin plus 2 weeks of oral doxycycline. In the ambulatory arm, the single dose of cefoxitin probably had little impact on anaerobic bacteria, whereas in the hospitalized arm patients received 48 hours of anaerobic therapy. No superiority was noted for either antimicrobial regimen, calling into question the need for anaerobic therapy in women with mild-to-moderate PID. In a recent editorial, Eschenbach also questioned a putative role for anaerobes in the pathogenesis of mild-to-moderate acute PID and suggested that although anaerobes may be present in the fallopian tubes, their role in the infectious process is not entirely clear [69].

However, concern remains about the importance of anaerobes in the pathogenesis and treatment of acute PID. Failing to provide anaerobic coverage in PID treatment regimen is problematic because there is limited data in support of the efficacy of such an approach. Hopefully, additional studies will address this issue and provide further insight into the role of anaerobes in PID.

Although recent reviews of PID treatment trials have noted that most antibiotic regimens, with the exception of the doxycycline and metronidazole regimen, result in fairly similar excellent clinical and microbiologic (primarily cervical *N. gonorrhoeae* and *C. trachomatis*) cure rates [17, 18, 63, 64], the search continues for treatment regimen(s) that optimize prevention of infertility, ectopic pregnancy, chronic pelvic pain, and recurrent infection. Three major determinants for preservation of post-PID fertility have been identified [3, 69]. These are (1) short duration of symptoms (<72 hours) prior to institution of therapy; (2) repetitive episodes of PID; (3) nongonococcal PID [16, 70, 71].

Duration of symptoms is the major determinant of subsequent infertility. Early diagnosis and treatment are crucial for preserving fertility and the effectiveness of antibiotic therapy is dependent upon the interval from the onset of symptoms to the initiation of treatment. In an updated analysis of the Lund, Sweden cohort of women with laparoscopically confirmed PID, Hillis and colleagues [71] demonstrated that women treated with ≥3 days of symptoms had a significantly greater infertility rate compared to those <3 days from symptom onset (19.7% versus 8.3%).

In their cohort of laparoscopically confirmed cases of PID, Westrom and colleagues reported that reinfection was an important predictor of subsequent tubal factor infertility [10]. In the most recent update of this cohort with 1,309 PID cases and 451 control patients who attempted to conceive, noted that the rate of infertility is directly proportional to both the number of episodes and severity of tubal inflammation seen at laparoscopy [11]. Each episode roughly doubles the rate of infertility; with one, two, or three or more episodes of PID infertility rates were 8.0%, 19.5%, and 40%, respectively. Among women with a single episode of PID, future fertility was associated with the severity of PID (at laparoscopy) ranging from 0.6% with mild disease to 6.2% and 21.4% for moderate and severe PID, respectively.

Studies based on the Swedish cohort [16, 70] have also demonstrated that women with chlamydial PID and nongonococcal nonchlamydial PID fared more poorly after treatment than those with gonococcal PID. Most likely for chlamydial PID, it is the delayed commencement of treatment associated with mild slow onset of symptoms. Nongonococcal nonchlamydial PID is more often associated with severe PID which is associated with a worse prognosis for future fertility.

## 4. Antimicrobial Treatment Regimens

Despite the controversy regarding the role of anaerobic bacteria and *M. genitalium* in the pathogenesis of acute PID, the polymicrobic nature of PID is widely acknowledged [1, 2]. As a consequence, PID is treated with antibiotics which provide coverage against a broad spectrum of potential pathogens. In 2010 the Center for Disease Control and Prevention updated their Guidelines for treatment of acute PID (Tables 2 and 3). According to the CDC 2010 guidelines, PID treatment regimens must provide empiric, broad spectrum coverage of likely pathogens [1]. These guidelines recommend that all treatment regimens should be effective against *N. gonorrhoeae* and *C. trachomatis* even in the presence of negative endocervical screening for these organisms. Although the CDC notes that the need to eradicate anaerobes from women with PID has not been definitively determined, as reviewed above, they suggest that until regimens without adequate coverage for anaerobes have been shown to prevent long-term sequelae as successfully as those that include anaerobic coverage, coverage of anaerobes should be considered in the treatment of acute PID.

As noted by the CDC [1] multiple randomized clinical treatment trials have demonstrated efficacy of both parenteral and oral regimens. In Table 4, the short-term clinical and microbiologic efficacy of oral and parenteral treatments regimens for PID are summarized. After excluding the metronidazole-doxycycline regimen (clinical and microbiologic cure rates 75% and 71%, resp.), the pooled clinical cure rates ranged from 88% to 99%, and the pooled microbiologic cure rates ranged from 89% to 100%. It is important that empiric treatment be initiated as soon as a presumptive diagnosis of acute PID is made because prevention of long-term sequelae is determined to a large extent by early administration (<72 hours) of appropriate antimicrobial therapy [1]. In addition, selection of a treatment regimen should consider availability, cost, patient acceptance, and antimicrobial acceptability [1, 72].

Because parenteral antibiotics do not necessarily require hospitalization, antibiotic regimens for the treatment of acute PID are categorized as follows:

1. regimens requiring more than a single parenteral dose as initial therapy are “parenteral” and
2. regimens that are primarily oral with or without an initial single parenteral dose are considered “oral.”

### 4.1. Parenteral Treatment

As noted in Table 4, several parenteral antimicrobial regimens have excellent short-term clinical and microbiological efficacy. Most of the literature supports the combination of (1) cefoxitin or cefotetan plus doxycycline and (2) clindamycin plus gentamicin. These two regimens remain the parenteral regimens recommended by the CDC for the treatment of PID. However, cefotetan is not currently marketed in the United States.

According to the CDC, there is limited data available supporting a role of other second or third generation parenteral cephalosporins (e.g., ceftizoxime, cefotaxime, or ceftriaxone) as effective therapy for acute PID and/or replacements for cefotetan or cefoxitin [1]. Moreover, these antimicrobial agents are less active against anaerobic bacteria than cefoxitin or cefotetan.

Intravenous infusion of doxycycline frequently causes pain and, thus, doxycycline should be administered orally whenever possible. Fortunately, oral and intravenous administration of doxycycline provide similar bioavailability [1].

With parenteral regimen A, parenteral therapy can be discontinued 24 hours after clinical improvement occurs [1]. However, oral doxycycline (100 mg twice a day) should be continued to complete a 14-day course of therapy. In cases involving tuboovarian abscess, either clindamycin (450 mg orally four times a day) or metronidazole (500 mg orally every 6 hours) should be used for continued therapy in order to provide more effective coverage against anaerobic bacteria.

There is concern over the increasing resistance of anaerobes, especially the *Bacteroides fragilis* group, to clindamycin [73, 74]. However, based on multiple clinical studies and extensive successful results with clindamycin containing regimens, clindamycin remains as a component in one of the recommended parenteral treatment regimens in both the CDC [1] and European [2] guidelines for treatment of PID.

Single dose gentamicin has not been evaluated for the treatment of acute PID. However, it is efficacious in the treatment of other pelvic and abdominal infections and is an option in parenteral regimen B. With this regimen, parenteral therapy may be discontinued 24 hours after clinical therapy. While the CDC suggests that either doxycycline 100 mg orally twice a day or clindamycin 450 mg orally four times a day to complete a total of 14 days of therapy may be used [1], in the author's opinion clindamycin oral therapy is preferred because of its better anaerobic coverage. In the presence of severe PID, especially tuboovarian abscess, clindamycin continued therapy is recommended by the CDC [1].

As noted by Walker and Wiesenfeld [17], there has been renewed interest in alternative agents, particularly ampicillin-sulbactam for anaerobic coverage. Unlike clindamycin, this agent has not been associated with selective pressure for microbial resistance. In addition, ampicillin-sulbactam is effective for *N. gonorrhoeae*. To provide adequate coverage for *C. trachomatis*, concomitant administration of doxycycline is recommended. Following clinical improvement, oral therapy with doxycycline 100 mg twice a day to complete 14 days of therapy should be continued. With severe disease, especially TOA, metronidazole 500 mg orally four times daily should be commenced as well.

While not included in the CDC 2010 recommended or alternative regimens for the treatment of PID, several factors have led clinicians to use azithromycin for the treatment of acute PID. These include widespread use in treating chlamydial infection, enhanced compliance due to its long half-life, and studies demonstrating the anti-inflammatory effects of macrolide antibiotics including azithromycin which appear to enhance host defense mechanisms and restrict local inflammation [17, 18, 75, 76]. A randomized clinical trial in the United Kingdom among 300 women with laparoscopically confirmed PID demonstrated excellent short-term clinical care rates with azithromycin monotherapy for one week (500 mg IV daily for one or two days followed by 250 mg for 5-6 days) or in combination with a 12 day course of metronidazole [77]. The microbiologic cure rates were also excellent (>95%) for *N. gonorrhoeae, C. trachomatis, M. hominis,* and anaerobes with these regimens. However, there was a large dropout rate with only one-third of the patients completing the study per protocol which, as noted by Haggerty and Ness [18] reduced the validity and generalizability of the microbiological cure evaluation. In addition, the anaerobic bacteria were only recovered from 27 (9%) of the patients, a rate substantially lower than noted in other studies.

The 2007 European guideline for the management of pelvic inflammatory disease contains similar recommendations [2].

As alternative regimens, the European guideline suggests i.v. ofloxacin 400 mg twice daily plus i.v. metronidazole 500 mg three times daily for 14 days or i.v. ciprofloxacin 200 mg twice daily plus i.v. (or oral) doxycycline 100 mg twice daily plus i.v. metronidazole 500 mg three times daily [2]. Because anaerobes are probably of relatively greater importance in patients with severe PID and some studies have demonstrated good clinical response without the use of metronidazole, the European guideline suggests that metronidazole may be discontinued in those patients with mild or moderate PID who are unable to tolerate it [2]. They further note that ofloxacin and moxifloxacin should be avoided in patients who are at high risk of gonococcal PID due to increasing quinolone resistance by *N. gonorrhoeae.*

### 4.2. Oral Treatment

Over the past 20 years, a new paradigm has emerged with a dramatic shift from hospital-based parenteral antibiotic regimens to oral ambulatory-based regimens [6, 7]. Initially, this shift was largely driven by the emergence of managed care and other economic factors without the benefit of clinical studies demonstrating that oral therapy was as effective as parenteral regimens, especially for prevention of long-term sequelae.

The PEACH study has provided evidence supporting the use of oral regimens on an ambulatory basis for the treatment of mild and moderately severe acute PID [42, 78]. PEACH, the largest randomized clinical treatment trial of acute PID in the United States, compared inpatient parenteral therapy (intravenous cefoxitin and oral or intravenous doxycycline during ≥48-hour hospitalization followed by oral doxycycline to complete a 14 day course) with outpatient oral therapy (a single intramuscular dose of cefoxitin with doxycycline administration orally for 14-days). Of most significance, PEACH not only assessed short-term but also long-term outcomes for over 800 patients (398 inpatient and 410 outpatient) with mild-to-moderately severe PID. The short-term clinical cure rates at 30 days were excellent in both groups, with roughly 3% of women in each group requiring additional treatment. At a mean followup of 35 months, the pregnancy rates were 42.0% and 41.7% with the outpatient and inpatient regimens, respectively. Long-term outcomes including infertility, ectopic pregnancy, recurrent PID, and chronic pelvic pain were also similar in both groups. However, as emphasized by Haggerty and Ness, despite high rates of clinical cure and eradication of *N. gonorrhoeae* and *C. trachomatis*, the rates of infertility (17%), recurrent PID (14%), and chronic pelvic pain (37%) were disappointingly high [18].

While data from the PEACH study suggests that neither the route nor site of treatment administration affects short-term or long-term outcomes among women with mild-to-moderately severe acute PID [42, 78], higher rates of post treatment histologic endometritis were present among the women in the outpatient oral group. However, the clinical significance of this finding is not clear. In previous studies we have shown that ongoing subclinical PID (as defined by histologic acute endometritis) is frequently present in women with untreated lower genital tract infections [14], and that persistent endometrial infection with *C. trachomatis *[26] and anaerobes [22] may lead to subsequent tubal damage and increased infertility among women with inadequately treated acute PID. Similarly, among women enrolled in the PEACH study, 23 out of 56 (41%) with *M. genitalium *identified in either the cervix and/or endometrium at baseline had *M. genitalium *persistently identified 30 days following treatment (inadequate to treat this organism) [40]. Moreover, women with persistent endometrial *M. genitalium *were 4.5 times more likely to experience short-term treatment failure (i.e., histologic endometritis and persistent pelvic pain at the 30-day follow-up visit).

As noted by the CDC [1], outpatient oral therapy can be considered for treatment of women with mild-to-moderately severe acute PID. The oral regimens listed in Table 3 provide coverage against the major etiologic agents of acute PID. Which of the cephalosporins is the optimum selection is unclear [1]. On the one hand cefoxitin has better anaerobic coverage, while ceftriaxone has better coverage against *N. gonorrhoeae*. The dose of ceftriaxone was increased to 250 mg IM in the 2010 CDC guidelines [1]. The extent of efficacy against anaerobic bacteria with a single dose of cefoxitin is questionable. However, in the PEACH study single dose cefoxitin was effective in obtaining clinical response [42, 78]. The CDC [1] and Walker and Wiesenfeld [17] have noted that theoretical limitations in coverage of anaerobes by recommended cephalosporins may require addition of metronidazole to the oral treatment recommendations. Addition of metronidazole to the oral regimens is the author's preferred approach. In addition, metronidazole will effectively treat bacterial vaginosis, which as noted above is frequently associated with PID. There is no published data on the use of oral cephalosporins for treatment of acute PID [1].

Information regarding alternative oral (outpatient) regimens is quite limited. Several alternative regimens have been the subject of at least one clinical trial and contain broad spectrum coverage [1]. These include (1) amoxicillin/clavulanic acid and doxycycline [79] and (2) Azithromycin monotherapy [77] or a combination of ceftriaxone 250 mg IM single dose with azithromycin 1 gram orally once a week for two weeks [80]. If one of these alternative regimens is selected, the CDC suggests the addition of metronidazole should be considered to cover anaerobic bacteria which are suspected as etiologic agents in PID and to effectively treat concomitant BV [1].

With the emergence of quinolone-resistant *N. gonorrhoeae*, regimens that include a quinolone agent are no longer recommended by the CDC for treatment of acute PID [1]. They note that in situations where single dose parenteral cephalosporin is not feasible, use of fluoroquinolones (levofloxacin 500 mg orally once a day or ofloxacin 400 mg orally twice a day for 14 days) with or without metronidazole (500 mg twice daily for 14 days) can be considered if community prevalence and individual risk for gonorrhea are low [1]. If this approach is selected, the CDC stresses that diagnostic tests for *N. gonorrhoeae* must be performed prior to initiating treatment [1]. Culture is the preferred test. If *N. gonorrhoeae* is detected, treatment should be based on the results of antimicrobial susceptibility. With quinolone-resistant *N. gonorrhoeae* or if susceptibility cannot be assessed (e.g., nucleic acid amplification test) use of a parenteral cephalosporin is recommended [1]. If use of a cephalosporin is not feasible, azithromycin 2 grams as a single dose can be added to a quinolone-based PID treatment regimen [1].

Patients treated with an oral regimen should demonstrate substantial clinical improvement within three days following initiation of treatment [1]. Clinical improvement is determined by defervescence, reduction in direct or rebound abdominal tenderness, and/or reduction in uterine, adnexal, and cervical motion tenderness. When patients fail to improve within this window, hospitalization is usually required for additional diagnostic tests (e.g., rule out TOA), parenteral antibiotic therapy, and/or surgical intervention [1].

### 4.3. Hospitalization for Treatment of Acute PID

While in the past, and to a lesser extent today, some clinicians have recommended that all patients with PID be hospitalized for parenteral antibiotics and bed rest, the PEACH study clearly demonstrated that in women with mild-to-moderately severe PID, outpatient oral therapy results in similar short- and long-term clinical outcomes as inpatient therapy [42]. As a result, the CDC notes that a decision regarding the need for hospitalization should be based on the judgment of the health-care provider and whether the patient meets any of the CDC suggested criteria for hospitalizations (Table 5). The European guideline concurs with these recommendations [2].

Limited studies have demonstrated that pregnant women with PID have high rates of fetal wastage and preterm delivery, supporting the appropriateness of hospitalization [81, 82]. Similarly, ample data supports hospitalization of women with TOAs in order to maximize antimicrobial dosing and close monitoring for early recognition of severe sepsis or of leaking/rupture of the abscess.

Several previous criteria for hospitalization have been removed from the current suggestions. The absence of data to support benefit from hospitalization for adolescent girls with PID led the CDC to not list adolescence among the criteria for hospitalization and to suggest that a decision to hospitalize adolescents with PID should be based on the same criteria used for older women [1]. In fact, subanalysis of the outcome data of the PEACH study stratified by age demonstrated that fertility outcomes of the adolescents were similar in the inpatient and outpatient treatment arms [78]. However, some clinicians continue to advocate that all adolescents and never pregnant young women should be hospitalized for treatment [83]. They argue that adolescence is a proxy for poor compliance, high-risk sexual activity, delayed care, and high antimicrobial failure rates.

Whereas the presence of HIV infection or immunosuppression has previously been an indicator for hospitalization and parenteral therapy, currently it is recommended that HIV-positive women with acute PID can be treated similarly to HIV-negative women. Although HIV-infected women who develop PID may have more severe clinical presentations and are more likely to have TOAs [84–86], there is no evidence to suggest that immunocompromised women benefit from hospitalization or parenteral therapy for uncomplicated PID [17, 87, 88].

### 4.4. Management of PID Associated with Intrauterine Contraceptive Device (IUD)

With the renewed interest in the IUD as a contraceptive choice for young women, PID will be seen in women using IUDs. As noted by Walker and Wiesenfeld, there does not exist any data to indicate that selection of treatment regimens should be influenced by the presence of an IUD [17]. In the past, clinicians generally removed IUDs to optimize the treatment of PID. This was primarily based on concerns that as a foreign body, removal of the IUD enhanced clinical response. Only a few studies have addressed this issue and the results are conflicting. In a small randomized study of 46 women in Sweden, Soderberg, and Lindgren [89] reported no differences in response to treatment whether the IUD was removed or left in place. On the other hand, Altunyurt and colleagues, in a randomized trial from Turkey, noted that clinical improvement (e.g., absence of pelvic pain, vaginal discharge, and pelvic tenderness) was more common in the group whose IUDs were removed [90]. If the provider elects to leave the IUD in place while PID is being treated, close clinical followup is important.

### 4.5. Management of Sex Partners

According to the CDC, male sex partners of women diagnosed with acute PID should be examined and treated if they had sexual contact with the patient during the preceding 60 days. If the last episode of sexual intercourse was > 60 days prior to onset of symptoms, the last sexual partner should be treated [1]. Women diagnosed with acute PID should refrain from sexual intercourse until treatment is completed and they and their partner(s) are asymptomatic. Sex partners of women with PID should be treated empirically with regimens effective against *N. gonorrhoeae* and *C. trachomatis *[1]. In those settings where only women are treated, arrangements should be undertaken to either provide care or appropriate referral for male sex partners [1]. Expedited partner treatment or enhanced patient referral are acceptable alternative approaches for the treatment of male partners of women who have PID with chlamydial or gonococcal infection [1].

## 5. Conclusion

Treatment strategies for women with acute PID should be based on the polymicrobial nature of this infection. The microorganisms recovered from the upper genital tract of women with acute PID include *N. gonorrhoeae, C. trachomatis*, and anaerobic and aerobic bacteria common to the endogenous vaginal flora and genital mycoplasmas, especially *M. genitalium*. Several antibiotic regimens are available which meet these requirements. Several parenteral antimicrobial regimens have been shown to provide very good short-term clinical and microbiological efficacy; these include clindamycin plus gentamicin, cefoxitin plus doxycycline, and cefotetan plus doxycycline.

Oral therapy for acute PID is currently the most commonly used approach, in response to both economic issues and the evidence from the PEACH study demonstrating that both short-term and long-term outcomes were similar for the oral and parenteral regimens. Due to the increased quinolone resistance of *N. gonorrhoeae*, choices of oral regimens are more limited. Ceftriaxone or cefoxitin demonstrated excellent short-term clinical and microbiological results. The addition of oral metronidazole to this regimen is suggested by some experts including this author to provide improved anaerobic coverage and at least to treat BV which is present in up to 70% of women with acute PID.

Currently regimens recommended by the CDC for the treatment of acute PID provide suboptimal antimicrobial activity against *M. genitalium *[40]. Mycoplasma lack a cell wall and thus are resistant to beta-lactam antibiotics (e.g., cefoxitin, cefotetan, ceftriaxone). Increased tetracycline resistance among *M. genitalium *has been reported [91]. In addition, *M. genitalium *is associated with persistent nongonococcal urethritis treated with tetracyclines [92]. Variable resistance to fluoroquinolones has been reported [93]. Recently, a newer fluoroquinolone, moxifloxacin, has demonstrated excellent activity against *M. genitalium *[91, 93]. This agent is one of the outpatient regimens recommended in the European guidelines [2]. While *M. genitalium *has demonstrated susceptibility to macrolides, azithromycin resistance has recently been reported [94].

## Figures and Tables

**Table 1:** Recovery of microorganisms from the upper genital tract of women with acute PID.

Study	Number of patients	*Chlamydia trachomatis*	*Neisseria gonorrhoeae*	Anaerobic and aerobic bacteria
Sweet [26–29]	380	68 (18%)	172 (45%)	267 (70%)
Wasserheit [30]	23	11 (44%)	8 (35%)	11 (45%)
Heinonen [31]	25	10 (40%)	4 (16%)	17 (68%)
Paavonen [32]	35	12 (34%)	4 (11%)	24 (69%)
Brunham [33]	50	21 (42%)	8 (16%)	10 (20%)
Soper [34]	84^a^	1 (1.2%)	32 (38%)	12 (13%)
	51^b^	6 (7.4%)	49 (98%)	16 (32%)
Hillier [35]	85^a^	3 (4%)	16 (19%)	43 (50%)
	178^b^	23 (13%)	44 (25%)	168 (94%)
	278^c^	27 (9.9%)	37 (13.4%)	170 (61%)
Haggerty [36]	45^c,d^	12 (26.5%)	15 (33.3%)	^ e^

Total	1234	194 (15.7%)	389 (31.5%)	770 (62%)

^a^Fallopian tube, cul-de-sac.

^b^Endometrial cavity.

^c^Clinically diagnosed acute PID.

^d^Histologic endometritis.

^e^Not available as total: anaerobic Gram-negative rods 31.7%; anaerobic Gram-positive cocci 22%; *Gardnerella vaginalis* 30.5%.

Reprinted with permission. Sweet [3].

**Table 2:** Parenteral treatment recommendations for acute pelvic inflammatory disease^a^.

Recommended regimen A

Cefotetan 2 g IV every 12 hours
Or
Cefoxitin 2 g IV every 6 hours
Plus
Doxycycline 100 mg orally or IV every 12 hours

Recommended regimen B

Clindamycin 900 mg IV every 8 hours
Plus
Gentamicin loading dose IV or IM (2 mg/Kg body weight)
followed by a maintenance dose (1–5 mg/Kg body weight)
every 8 hours.
A single daily dosing (3–5 mg/Kg) can be substituted

Alternative parenteral regimen

Ampicillin/sulbactam 3 g IV every 6 hours
Plus
Doxycycline 100 mg orally or IV every 12 hours

^a^CDC Sexually Transmitted Diseases Treatment Guidelines 2010 MMWR 2010 : 59 (no.-RR12): [63–67].

**Table 3:** Oral treatment recommendations for acute pelvic inflammatory disease^a^.

Recommended regimens
(1) Ceftriaxone 250 mg IM in a single dose
Plus
Doxycycline 100 mg orally twice a day for 10–14 days
With or without
Metronidazole 500 mg orally twice a day for 10–14 days
(2) Cefoxitin 2 g IM in a single dose and Probenecid 1 g orally
administered concomitantly as a single dose
Plus
Doxycycline 100 mg orally twice a day for 10–14 days
With or without
Metronidazole 500 mg orally twice a day for 10–14 days
(3) Other parenteral third generation cephalosporins
(e.g., ceftizoxime or cefotaxime) in a single dose
Plus
Doxycycline 100 mg orally twice a day for 10–14 days
With or without
Metronidazole 500 mg orally twice a day for 10–14 days

^a^CDC Sexually Transmitted Diseases Treatment Guidelines 2010 MMWR 2010 : 59 (no.-RR12).

**Table 4:** Clinical and microbiologic cure rates for pelvic inflammatory disease treatment regimens.

	Clinical cure			Microbiologic cure		
Regimen	Number of studies	Number of patients	Percent	Number of studies	Number of patients	Percent
Parenteral						
Clindamycin/aminoglycoside	11	470	92	8	143	97
Cefoxitin/doxycycline	9	836	95	7	581	96
Cefotetan/doxycycline	3	174	94	2	71	100
Ciprofloxacin	4	90	94	4	72	96
Ofloxacin	2	86	99	2	50	98
Sulbactam-ampicillin/doxycycline	1	37	95	1	33	100
Metronidazole/doxycycline	2	36	75	1	7	71
Azithromycin	1	30	100	1	30	100
Azithromycin/metronidazole	1	30	97	1	30	97
Oral						
Ceftriaxone/probenecid/doxycycline	1	64	95	1	8	100
Cefoxitin/probenecid/doxycycline	3	212	90	3	71	93
Cefoxitin/doxycycline	4	634	94	4	493	95
Amoxicillin-clavulanic acid	2	35	100	2	35+	100
Ciprofloxacin/clindamycin	1	67	97	1	10	90
Ofloxacin	2	165	95	2	42+	100
Levofloxacin	1	41	85	1	9	89

Reprinted with permission from Walker and Sweet [64].

**Table 5:** Suggested criteria for hospitalization for treatment of acute PID^a^.

(i) Surgical emergencies (e.g, appendicitis) cannot be ruled out
(ii) Patient is pregnant
(iii) Patient does not respond clinically to oral antimicrobial
therapy
(iv) Patient unable to follow or tolerate outpatient oral regimen
(v) Patient has severe illness, nausea, vomiting, or high fever
(vi) Patient has a tuboovarian abscess

^a^CDC Sexually Transmitted Diseases Treatment Guidelines 2010 MMWR 2010 : 59 (no.-RR12).
